# Wearable Activity Tracker–Based Interventions for Physical Activity, Body Composition, and Physical Function Among Community-Dwelling Older Adults: Systematic Review and Meta-Analysis of Randomized Controlled Trials

**DOI:** 10.2196/59507

**Published:** 2025-04-03

**Authors:** Ran Li, Yangan Li, Lu Wang, Lijuan Li, Chenying Fu, Danrong Hu, Quan Wei

**Affiliations:** 1 Department of Rehabilitation Medicine Center and Institute of Rehabilitation Medicine, West China Hospital, Sichuan University Chengdu, Sichuan China; 2 Key Laboratory of Rehabilitation Medicine in Sichuan Province Chengdu, Sichuan China; 3 National Clinical Research Center for Geriatrics, West China Hospital, Sichuan University Chengdu, Sichuan China; 4 Aging and Geriatric mechanism laboratory, West China Hospital, Sichuan University Chengdu, Sichuan China

**Keywords:** wearable activity tracker, physical activity, older adult, systematic review, meta-analysis

## Abstract

**Background:**

The global aging population faces great challenges. Wearable activity trackers have emerged as tools to promote physical activity among older adults, potentially improving health outcomes. However, the effectiveness of such interventions on physical activity, body composition, and physical function among community-dwelling older adults remains debated.

**Objective:**

This study conducted a systematic review and meta-analysis to evaluate the impact of wearable activity tracker–based interventions on physical activity, body composition, and physical function among community-dwelling older adults.

**Methods:**

We searched the PubMed, Embase, Web of Science, and CENTRAL databases from inception until January 2025 to identify related randomized controlled trials. The outcomes were focused on physical activity (physical activity time, daily step count, and daily sedentary time); body composition (BMI and body fat); and physical function (timed up and go test and chair stand test). Subgroup analysis by different controls (usual care or conventional interventions) and different follow-ups (immediate or short term) were performed.

**Results:**

In total 23 trials with 4566 participants were eligible for analysis. Compared to usual care, there was lo- to moderate-certainty evidence that the wearable activity tracker–based interventions significantly increased physical activity time (standardized mean difference [SMD]=0.28, 95% CI 0.10-0.47; *P*=.003) and daily step counts (SMD=0.58, 95% CI 0.33-0.83; *P*<.001) immediately after intervention, while no significant improvements were observed in daily sedentary time (mean difference [MD]=−1.56, 95% CI −10.88 to 7.76; *I*^2^=0%; *P*=.74). These interventions were at least as effective as conventional interventions but did not show superiority. Compared with usual care, the interventions using wearable activity trackers only demonstrated a notable increase in daily step count over short-term follow-up (SMD=0.23, 95% CI 0.11-0.36; *P*<.001). As for body composition and physical function, there was low- to moderate-certainty evidence that the wearable activity tracker–based interventions did not have a greater impact on BMI (MD=0.40, 95% CI −0.08 to 0.89; *P*=.11), body fat (MD=0.67, 95% CI −0.54 to 1.87; *P*=.28), the timed up and go test (MD=0.14, 95% CI −0.87 to 1.16; *P*=.78), or the chair stand test (SMD=−0.31, 95% CI −0.62 to 0; *P*=.05).

**Conclusions:**

This systematic review and meta-analysis indicate that wearable activity tracker–based interventions were effective in enhancing physical activity with low to moderate certainty, but did not significantly impact body composition or physical function, with low to moderate certainty, among community-dwelling older adults, particularly immediately after intervention. This intervention showed a more pronounced impact when compared to usual care, rather than to conventional interventions, with low to moderate certainty. It is important to note that this intervention showed moderate-certainty evidence toward improving daily step count, supporting its sustained impact during short-term follow-up.

**Trial Registration:**

PROSPERO CRD42024516900; https://www.crd.york.ac.uk/PROSPERO/view/CRD42024516900

## Introduction

### Background

As the global population ages, the number of individuals aged ≥60 years is expected to double by 2050 to about 2 billion. Advances in diet, lifestyle, education, and health care have increased life spans [1]. However, healthy, disease-free years have not increased at the same pace [2,3]. This gap leads to more chronic diseases and a lower quality of life among older adults [4,5]. Regular physical activity is essential for preventing chronic conditions, enhancing cognitive function, and improving overall well-being, particularly in older populations [6-8]. However, community-dwelling older adults face a higher risk of physical inactivity, especially after the COVID-19 pandemic [9,10]. Therefore, effective strategies are urgently needed to encourage sustained physical activity in this group.

Wearable activity trackers, known for their user-friendly design [11] and affordability [12], have emerged as innovative tools for monitoring and promoting individual physical activity. These devices provide real-time, objective feedback on physical activity and body composition, such as step count, weight data, and energy expenditure, enabling users to track their progress and make informed behavioral adjustments [13-15]. The impact of wearable activity trackers on promoting sustained physical activity can be better understood through theoretical models of behavior change. Self-determination theory emphasizes the role of intrinsic motivation and autonomy [16], with wearable activity trackers enhancing competence by providing tangible progress feedback and reinforcing self-efficacy and goal attainment. However, long-term adherence often requires additional support, such as personalized goal setting and social interaction [16,17]. Social cognitive theory further explains how wearable trackers influence behavior through self-regulation, observational learning, and reinforcement [18]. Real-time feedback helps to assess current activity levels against predefined goals, strengthening self-monitoring and self-efficacy, while social features such as peer comparisons enhance motivation through observational learning and positive reinforcement. However, their true potential often lies in how they are integrated into broader behavioral interventions. A recent trial indicated that wearable activity trackers might be more effectively used as a medium for delivering structured intervention strategies, rather than serving as standalone tools [19]. Wearable activity tracker–based interventions leverage the unique capabilities of these devices to provide personalized goal setting and motivational activation [20] and are often designed to promote behavior change and increase adherence, particularly in boosting physical activity and physiological outcomes [21]. Unlike conventional interventions that may rely on in-person counseling or structured exercise programs, wearable activity tracker–based interventions provide continuous monitoring and feedback, allowing for integration into daily routines. Many are further enhanced by telehealth platforms or mobile apps, offering hybrid approaches that facilitate remote support and communication with health care providers [22,23]. However, the extent to which wearable activity tracker–based interventions can drive changes in physical activity, particularly for their potential to integrate into the daily lives of community-dwelling older adults, remains a subject of ongoing research [24-26].

Several systematic reviews have explored the effectiveness of wearable activity trackers and related interventions in various populations, including older adults. For example, 2 systematic reviews suggested that wearable activity tracker–based interventions had a positive effect on improving physical activity levels among older adults [27,28]. Similarly, another recent systematic review revealed that wearable activity trackers significantly increased daily steps and physical activity among older adults, particularly when combined with other interventions [29]. However, some reviews included older adults in hospital settings, which may confound findings due to varying baseline activity levels and distinct health needs. Moreover, a key gap in literature is the limited focus on community-dwelling older adults, a population that is particularly relevant for real-world interventions. Unlike those in institutional settings, community-dwelling older adults have greater opportunities to integrate interventions into their daily routines, making them an important target for interventions aimed at promoting healthy aging. In addition, variations in follow-up periods and control group types across studies complicate the interpretation of results, as these factors can influence observed outcomes [30-34]. Finally, while previous reviews primarily focused on physical activity outcomes, they often overlooked broader impacts on body composition and physical function, which are vital indicators of overall health and independence for older adults.

### Objectives

To address this current evidence gap, this systematic review and meta-analysis aimed to synthesize existing evidence from randomized controlled trials (RCTs) to ascertain the impact of interventions using wearable activity trackers on physical activity, body composition, and physical function among community-dwelling older adults, with a particular focus on the effects of follow-up periods and varying control conditions.

## Methods

### Design

This systematic review and meta-analysis followed the PRISMA (Preferred Reporting Items for Systematic Reviews and Meta-Analyses) [35] and PRISMA 2020 guidelines [36] and was performed following a protocol registered in PROSPERO (International Prospective Register of Systematic Reviews; CRD42024516900).

### Search Strategy

Two reviewers independently searched the PubMed, Embase, Web of Science, and CENTRAL databases from inception until January 2025 without language restrictions. The following terms were searched as keywords: “activity trackers,” “wearable tracker,” “pedometer,” “older,” “elder” and “randomized controlled trial.” The comprehensive search methodology is present in Multimedia Appendix 1. The reference lists of the included studies, along with those of previous systematic reviews, were screened for additional potentially eligible studies.

### Study Selection

All studies were systematically screened by 2 independent reviewers at each stage of the evaluation process, including title, abstract, and full-text assessment. When there was a disagreement, 2 more reviewers engaged in a thoughtful conversation until a consensus was reached.

This systematic review included studies concerning the effects of wearable activity trackers on older adults. The inclusion criteria were as follows: (1) RCTs of parallel groups; (2) participants of community-dwelling older adults; (3) participants aged ≥55 years, or the average or median participant age was ≥55 years; (4) wearable activity trackers alone or in combination with other components as an intervention; (5) studies reported on ≤1 outcome measured physical activity, body composition, and physical function. The exclusion criteria were as follows: (1) protocols, reviews, case reports, and conference abstracts; (2) older adults participants who were hospitalized; (3) intervention with wearable activity tracker as control; (4) sample size <10.

### Data Extraction

Two reviewers independently extracted the main information for the included studies using a standard extraction spreadsheet on Microsoft Excel. A third reviewer was consulted if the initial reviewers disagreed. The detailed characteristics of the selected studies were summarized, which included study characteristics (author, year of publication, country, study design, sample size, outcome measurement, and follow-up); population characteristics (age and sex); intervention characteristics (intervention type, duration, and device type). When available, data on physical activity, body composition, and physical function were also extracted.

### Quality Assessment

All studies were appraised for methodological quality using the Physiotherapy Evidence Database (PEDro) scale. The PEDro scale score assesses the internal validity of RCTs with 10 scored items, including random allocation, concealed allocation, baseline comparability, participant blinding, therapist blinding, assessor blinding, adequate follow up, intention-to-treat analysis, between-group statistical comparison, and point and variability measure [37,38]. Items are given a score of either present (1) or absent (0). A summation is used to determine the score out of 10, with a score of ≥6 being regarded as high quality.

The evidence quality was evaluated using the Grading of Recommendations, Assessment, Development, and Evaluation approach, with 4 ratings: high, moderate, low, and very low [39]. RCTs are rated as having high quality at first and are subsequently downgraded due to risk of bias (trials with low methodological quality: PEDro score <6), imprecision (fewer than 300 participants for each outcome), inconsistency (large heterogeneity between the trials *I*^2^>50%), indirectness (indirect comparisons between populations, interventions, or outcomes), and publication bias (funnel plot asymmetry if ≥10 trials are included in meta-analysis) [40-44].

Before conducting the meta-analysis, the methodological quality and evidence quality assessment were independently performed by 2 reviewers. Discrepancies were resolved by consensus with a third researcher.

### Statistical Analysis

This meta-analysis was conducted using Review Manager (version 5.4; Cochrane Collaboration). To evaluate the effectiveness of wearable activity trackers in older adults, we conducted a meta-analysis by pooling the means and SDs for outcomes of interest from each study. Mean differences (MDs) with the 95% CIs were calculated using the inverse variance method when the continuous outcomes were evaluated with the same scale, while standardized MDs (SMDs) with 95% CIs were calculated when continuous outcomes were evaluated with different scales. Statistical differences according to meta-analysis were identified as those for which *P*<.05. The chi-square test and inconsistency (*I*^2^) were used to calculate statistical heterogeneity. The fixed-effect model was used when *I*^2^<50%; otherwise, the random-effect model was used. When ≥2 methods of assessing an outcome were used in 1 study, either the method defined as being the gold standard or the method with high validity and reliability was used. Publication bias was assessed using funnel plots and Egger test where ≥10 studies were included in the meta-analysis [45].

### Subgroup Analysis

Subgroup analysis by different controls and different follow-ups were performed on outcomes. If there were both active and passive control groups in one trial, we handled the treatment with each control as an independent comparison in subgroup analysis to account for these articles. The passive control group received usual care, which consisted of standard care and self-management guidance. In contrast, the active control group underwent conventional interventions, which included a blend of behavior change techniques, tailored exercises, and prescribed physical activity, but without the use of wearable activity trackers. Controls that included the use of wearable activity trackers were not considered for this comparison to ensure a fair and rigorous assessment of the effectiveness. When multiple follow-up data points were available, the data collected immediately following intervention completion and at the final follow-up were selected for subgroup analysis. These 2 time points were designated as representing the immediate postintervention outcomes and the short-term outcomes, respectively.

## Results

### Study Selection and Characteristics

**Figure 1:**
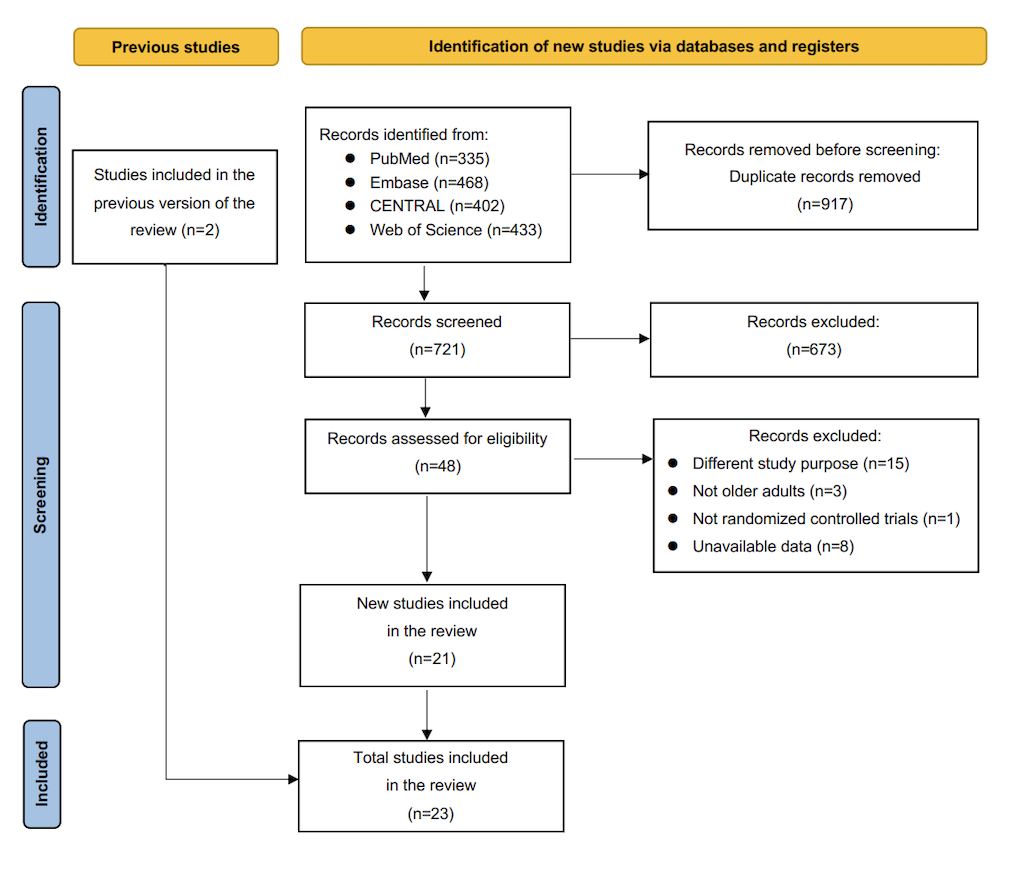
A flowchart showing the study selection process. RCT: randomized controlled trial.

The initial search procedure yielded 1638 records in total, with an additional 2 articles identified through manual reference checks of relevant articles. After removing duplicate citations, 721 studies remained for title and abstract screening, 48 of which were considered potentially eligible for full-text review. In total, 23 eligible trials [30-34,46-62] were selected for this systematic review and meta-analysis (Figure 1).

All studies used an RCT design, with 7 (30%) using a 3-arm parallel-group design [30,46,47,49,54,58,59]. The sample size ranged from 34 to 1023, with 4566 older adults across all studies. The mean age of participants ranged from 58 (SD 5.8) to 80 (SD 6.8) years. Of the included articles, one focused exclusively on a female population [52]. A total of 6 (26%) studies of wearable activity tracker–based interventions used the Yamax pedometer [48-50,56,60], 5 (22%) studies used the Fitbit pedometer [31-33,46,54], 2 (9%) studies used the Kens Lifecorder pedometer and accelerometer [51,52], 2 (9%) studies used the Jawbone Up [47,53], 2 (9%) studies used the Omron pedometer [58,59], 1 (4%) trial used the Garmin Vivofit 4 [63], and 1 (4%) trial used the Polar Loop [34]. In total, 4 (17%) trials did not report the type of wearable activity tracker used in the intervention [30,55,57,61]. A detailed overview of the study characteristics and the demographic profile of the participants is presented in Table 1.

**Table 1:** Characteristics of the included studies.

Study	Country	Study design	Sample size (at baseline)	Sex, n (%)	Age (y)	Interventions	Duration	Trackers	Outcomes	Follow-up
Alley et al [46], 2022	Australia	3-arm RCT^a^	Total (n=243); INT^b^ (n=78); CON1 (n=96); CON2 (n=69)	Male, 52 (21); Female, 191 (79)	INT1 (mean 69.88, SD 4.1); INT2 (mean 69.12, SD 4.93); CON^c^ (mean 68.84, SD 3.85)	INT1: computer-tailored advice, action-planning tool, and exercise library that synced Fitbit activity tracker with the website to measure physical activity; CON1: same as INT1 but without tracker; CON2: usual care	12 wk	Fitbit	Physical activity	6 wk, 12 wk, and 24 wk
Armit et al [30], 2009	Australia	3-arm RCT	Total (n=136); INT (n=45); CON1 (n=45); CON2 (n=46)	Male, 54 (40); Female, 82 (60)	Mean 58 (SD 5.8)	INT1: behavior change advice with goal setting and self-monitoring focusing on a pedometer; CON1: behavior change advice from an exercise scientist; CON2: usual care plus brief advice	12 wk	—^d^	Physical activity, BMI, blood pressure, and cardio-respiratory fitness	12 wk and 24 wk
Bailey et al [63], 2024	England	2-arm RCT	Total (n=60); INT (n=30); CON(n=30)	Male, 20 (33); Female, 40 (67)	INT (mean 75, SD 7); CON (mean 74, SD 6)	INT: multicomponent interventions with tailored feedback, an education workbook, health coaching, peer support, and a wearable device; CON: usual care	24 wk	Garmin Vivofit 4	Physical activity; BMI; and body composition	24 wk
Brickwood et al [47], 2021	Australia	3-arm RCT	Total (n=117); INT1 (n=37); CON1 (n=38); CON2 (n=42)	Male, 42 (36); Female, 75 (64)	INT1 (mean 72.3, SD 7); INT2 (mean 72.8, SD 7); CON (mean 71.9, SD 6)	INT1: home-based exercise program plus daily feedback via app based on the data from the tracker and weekly feedback via text message; CON1: home-based exercise program plus a physical activity counseling phone call; CON2: usual care	48 wk	Jawbone UP24	Physical activity, body weight, BMI, blood pressure, 10-TSTST^e^, TUG^f^ test, 6MWT^g^, modified SWT^h^, and SF-36^i^	12 wk, 24 wk, and 48 wk
Croteau et al [48], 2007	United States	2-arm RCT	Total (n=179); INT (n=95); CON (n=84)	Male, 32 (22); Female, 115 (78)	INT (mean 74.4, SD 9.1); CON (mean 71.2, SD 8.2)	INT: social cognitive theory-based intervention that consisted of counseling, pedometer usage, and self-monitoring; CON: usual care	12 wk	Yamax Digi-Walker SW-200 pedometer	Physical activity	12 wk and 24 wk
Harris et al [50], 2015	England	2-arm RCT	Total (n=298); INT (n=150); CON (n=148)	Male, 138 (46); Female, 160 (54)	Range 60-75	INT: behavior change techniques, pedometer step count and accelerometer PA^j^ intensity feedback, an individual PA diary, and 4 primary care nurse physical activity consultations; CON: usual care	12 wk	Yamax Digi-Walker SW-200 pedometer	Physical activity, 15-GDS^k^, 4-item FEAR^l^ score, 4-item self-reported pain score, adverse events, BMI, and body fat	12 wk and 48 wk
Harris et al [49], 2017	England	2-arm RCT	Total (n=1023); INT (n=339); CON1 (n=346); CON2 (n=338)	Male, 367 (36); Female, 656 (64)	Range 45-75	INT: behavior change techniques, pedometer step count, and PA diary; CON1: behavior change techniques, pedometer step count, PA diary and plan plus 3 individually tailored practice nurse PA consultations; CON2: usual care	12 wk	Yamax Digi-Walker SW-200 pedometer	Physical activity, 15-GDS, 4-item FEAR score, 4-item self-reported pain score, BMI, body fat, waist circumference, and adverse events	12 wk and 48 wk
Kawagoshi et al [51], 2015	Japan	2-arm RCT	Total (n=27); INT (n=15); CON (n=12)	Male, 24 (89); Female, 3 (11)	INT (mean 75, SD 9); CON (mean 74, SD 8)	INT: pulmonary rehabilitation and feedback from pedometer use; CON: pulmonary rehabilitation only	48 wk	Kens Lifecorder EX pedometer	Physical activity, pulmonary function, respiratory muscle strength, quadriceps femoris muscle force, 6MWT, MRC^m^ dyspnea scale, BODE^n^ index, CRQ^o^	48 wk
Koizumi et al [52], 2009	Japan	2-arm RCT	Total (n=68); INT (n=34); CON (n=34)	Female, 27 (100)	INT (mean 66, SD 4); CON (mean 67, SD4)	INT: pedometer-based behavioral change interventions; CON: usual care	12 wk	Kenz Lifecorder accelerometer	Physical activity and 12MWT^p^	12 wk
Liu et al [31], 2021	China	2-arm RCT	Total (n=40); INT (n=22); CON (n=18)	Male, 6 (15); Female, 34 (85)	INT (mean 72.1, SD 3.7); CON (mean 80.4, SD 6.83)	INT: group-based exercise intervention consisted of tracker-based training and physical training adopted from behavioral change techniques; CON: only physical training involving behavioral change techniques plus a health talk	14 wk	Fitbit	Physical activity, 30CS^q^ test, TUG test, 2MWT^r^, FFI^s^ score, 9-item CSEE^t^, 19-item C-BREQ-2^u^, and adverse event	14 wk, 26 wk, and 38 wk
Lyons et al [53], 2017	United States	2-arm RCT	Total (n=40); INT (n=20); CON (n=20)	Male, 6 (15); Female, 34 (85)	INT (mean 61.25, SD 5); CON (mean 61.7, SD 6.26)	INT: intervention combining a wearable physical activity monitor, tablet device, and telephone counseling; CON: usual care	12 wk	Jawbone UP24	Physical activity, 6MWT, body fat, and adverse event	12 wk
Muellmann et al [54], 2019	Germany	3-arm RCT	Total (n=589); INT1 (n=198); CON1 (n=211); CON2 (n=180)	Male, 230 (43); Female, 299 (57)	INT1 (mean 69.6, SD 3.2); INT2 (mean 69.6, SD 3.4); CON (mean 69.8, SD 3.2)	INT1: web-based interventions based on self-regulation theory and principles of behavior change with a physical activity tracker; CON1: same as INT1 but with a web-based PA diary; CON2: usual care	10 wk	Fitbit	Physical activity	12 wk
Mutrie et al [55], 2012	England	2-arm RCT	Total (n=41); INT (n=20); CON (n=21)	Male, 13 (32); Female, 28 (68)	INT (mean 71.6, SD 6); CON (man 70, SD 4.3)	INT: pedometer-based walking program in combination with physical activity consultations; CON: usual care	12 wk	—	Physical activity, SF-36, PANAS^v^, PMES-OA^w^, UCLA^x^ Loneliness Scale, and adverse event	12 wk and 48 wk
Nishiguchi et al [56], 2015	Japan	2-arm RCT	Total (n=48); INT (n=24); CON (n=24)	Male, 26 (54); Female, 22 (46)	INT (mean 73, SD 4.8); CON (mean 73.5, SD 5.6)	INT: group training sessions and pedometer-based walking exercise; CON: usual care	12 wk	Yamax Power Walker EX-300 pedometer	Physical activity, 10MWT^y^, TUG, 5CS^z^ test, WMS-R^aa^, TMT^ab^, MMSE^ac^, whole-brain imaging, and adverse event	12 wk
Oliveira et al [33], 2024	Australia	2-arm RCT	Total (n=605); INT (n=290); CON (n=315)	Male, 180 (30); Female, 425 (70)	INT (mean 74, SD 7.5); CON (mean 75, SD 8.5)	INT: physical activity plan with the health coach, received an activity monitor (Fitbit or pedometer); CON: a 12-month nutrition program with a booklet about healthy nutrition and access to telephone-based health coaching focused on healthy eating	48 wk	Fitbit	Physical activity, EQ-5D-3L^ad^, FESI^ae^, PANAS, LLFDI^af^, and adverse event	24 wk and 48 wk
Oliveira et al [32], 2019	Australia	2-arm RCT	Total (n=131); INT (n=64); CON (n=67)	Male, 38 (29); Female, 93 (71)	INT (mean 71, SD 6); CON (mean 72, SD 7)	INT: 1 physiotherapist visit, fortnightly telephone-based health coaching, a pedometer, tailored fall prevention advice, and a fall prevention brochure; CON: fall prevention brochure only	24 wk	Fitbit	Physical activity, falls rate, COMPAS-W^ag^, EQ-5D-5L, VAS^ah^, Goal Attainment Scale, BMI, FESI, PANAS, modified GES^ai^, and adverse event	12 wk, 24 wk, and 48 wk
Patel et al [57], 2013	Australia	2-arm RCT	Total (n=225); INT (n=116); CON (n=109)	Male, 102 (45); Female, 123 (55)	≥65	INT: a pedometer to accumulate steps through prescribed activity with telephone-based counseling; CON: prescribed activity for a set period per day (eg, a 30-min session of walking or swimming) with telephone-based counseling	12 wk	—	Physical activity, 15-GDS, and SF-36	12 wk and 48 wk
Rowley et al [58], 2019	United States	3-arm RCT	Total (n=170); INT (n=57); CON1 (n=62); CON2 (n=51)	Male, 35 (21); Female, 135 (79)	INT1 (mean 67.4, SD 6.4); INT2 (mean 68.3, SD 7.1); CON ( mean 66.1, SD 4.9)	INT: tailored internet-mediated pedometer intervention; CON1: pedometer only intervention with 10,000 steps; CON2: usual care	12 wk	Omron HJ-720ITC pedometer	Physical activity	12 wk
Suboc et al [59], 2014	United States	3-arm RCT	Total (n=114); INT (n=34); CON1 (n=38); CON2 (n=42)	Male, 71 (66); Female, 36 (34)	INT1 (mean 63, SD 8); INT2 (mean 64, SD 7); CON (mean 62, SD 7)	INT: pedometer combined with interactive website intervention; CON1: pedometer only intervention; CON2: usual care	12 wk	Omron HJ-720ITC pedometer	Physical activity, endothelial function, and vascular compliance	12 wk
Suorsa et al [34], 2022	Finland	2-arm RCT	Total (n=231); INT (n=117); CON (n=114)	Male, 40 (17); Female, 191 (83)	INT (mean 65.2, SD 1); CON (mean 65.2, SD 1.1)	INT: pedometer-based behavioral change interventions; CON: usual care	48 mo	Polar Loop 2	Physical activity and SF-36	12 wk, 24 wk, and 48 wk
Talbot et al [60], 2003	United States	2-arm RCT	Total (n=34); INT (n=17); CON (n=17)	Male, 8 (24); Female, 26 (76)	INT (mean 69.59, SD 6.74); CON (mean 70.76, SD 4.71)	INT: self-management education plus pedometer intervention; CON: self-management education only	12 wk	Yamax Digi-Walker SW-200 pedometer	Physical activity, muscular strength, 100-FTTWT^aj^, timed stair climb, timed chair rise, and McGill Pain Questionnaire	12 wk and 24 w
Yamada et al [62], 2012	Japan	2-arm RCT	Total (n=87); INT (n=43); CON (n=44)	Male, 40 (49); Female, 42 (51)	INT (mean 75.5, SD 5.9); CON (mean 75.8, SD 7.6)	INT: pedometer-based behavioral change interventions; CON: usual care	24 wk	Yamax Powerwalker EX-510	Physical activity, 10MWT, TUG test, FR^ak^ test, 5CS test, fall experience and fear of falling, and BIA^al^	24 wk
Yuenyongchaiwat and Akekawatchai [61], 2022	Thailand	2-arm RCT	Total (n=60); INT (n=30); CON (n=30)	Male, 34 (57); Female, 26 (43)	INT (mean 69.23, SD 6.71); CON (mean 71.93, SD 5.19)	INT: encouraging walking ≥7500 steps daily with a pedometer and resistance exercise with elastic TheraBand; CON: usual care	12 wk	—	Physical activity, sarcopenic assessments, 6MWT, and respiratory muscle strength	12 wk

^a^RCT: randomized controlled trial.

^b^INT: intervention group.

^c^CON: conventional group.

^d^Not available.

^e^10-TSTST: 10-time sit-to-stand test.

^f^TUG: timed up and go.

^g^6MWT: 6-minute walk test.

^h^SWT: shuttle walk test.

^i^SF-36: Short-Form 36-Item Health Survey.

^j^PA: physical activity.

^k^15-GDS: 15-item Geriatric Depression Score.

^l^FEAR: frequency of anxiety, enduring nature of anxiety, alcohol or sedative use, and restlessness or fidgeting.

^m^MRC: Medical Research Council.

^n^BODE: BMI, airflow obstruction, dyspnea, and exercise capacity index.

^o^CRQ: Chronic Respiratory Disease Questionnaire.

^p^12MWT: 12-minute walk test.

^q^30CS: 30 Chair to Stand Test.

^r^2MWT: 2-minute walk test.

^s^FFI: Fried Frailty Index.

^t^CSEE: Chinese self-efficacy for exercise scale.

^u^C-BREQ-2: Chinese version 2 of the Behavioral Regulation in Exercise Questionnaire-2.

^v^PANAS: positive and negative affect schedule.

^w^PMES-OA: Perceived Motor-Efficacy Scale for Older Adults.

^x^UCLA: University of California, Los Angeles.

^y^10MWT: 10 m walk test.

^z^5CS: 5 Chair to Stand Test.

^aa^WMS-R: Wechsler memory scale revised.

^ab^TMT: Trail-Making Test.

^ac^MMSE: Mini-Mental State Examination.

^ad^EQ-5D-3L: Self-report European quality of life-5 dimensions.

^ae^FESI: Falls Efficacy Scale International.

^af^LLFDI: Late Life Function and Disability Instrument.

^ag^COMPAS-W: composite scale of well-being.

^ah^VAS: Visual Analog Scale.

^ai^GES: Gait Efficacy Scale.

^aj^100-FTTW: 100-foot timed walk-turn-walk.

^ak^FR: functional reach test.

^al^BIA: bioelectrical impedance analysis.

### Methodological Quality

The total PEDro score varied from 3 to 8, with an average of 6. In total, 56% (13/23) of studies were classified as high quality. All studies met the criteria for random allocation and between-group comparison, as well as the calculation of point estimates and variability. However, none of the trials incorporated participant or therapist blinding, which is not typically feasible in this type of intervention. The methodological quality and reporting of the eligible trials are summarized in Multimedia Appendix 2 [30-34,46-63].

### Effects on Physical Activity

#### Physical Activity Time

**Figure 2:**
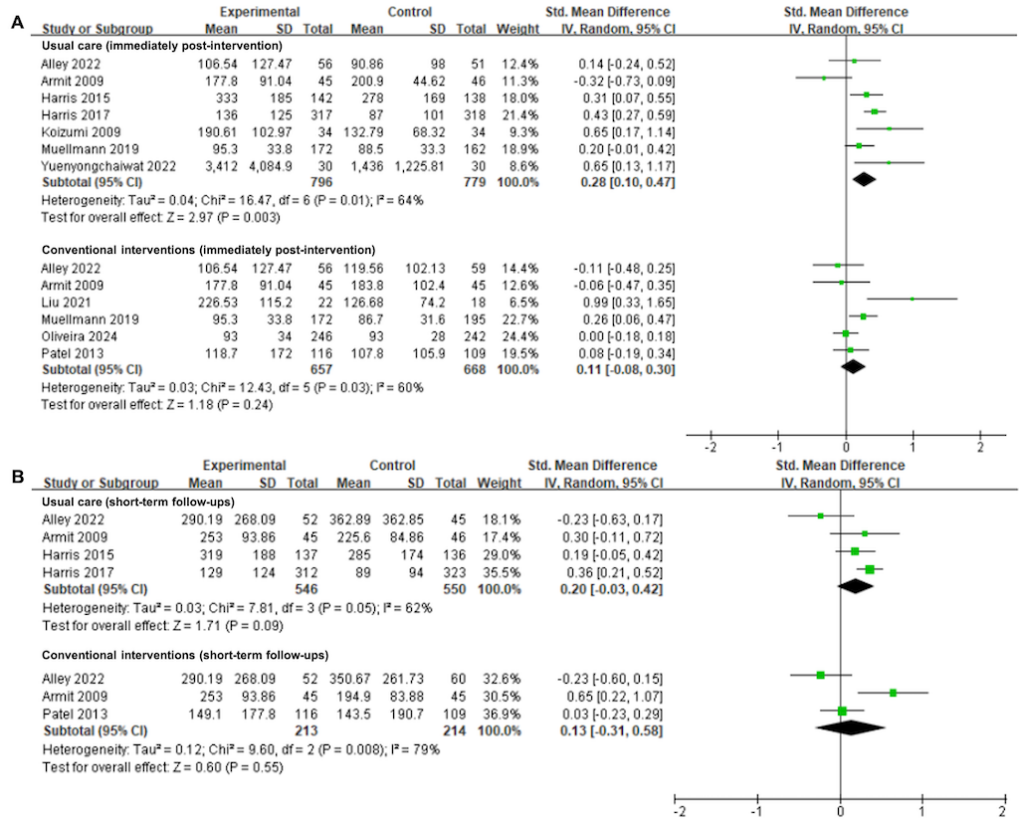
Forest plots for wearable activity tracker–based interventions compared with usual care and conventional interventions in physical activity time at (A) immediately after intervention and (B) short-term follow-ups. Green squares indicate standardized mean differences and mean differences, with larger squares reflecting greater weight; horizontal lines indicate 95% CI; and black diamonds indicate pooled effect estimates, with right and left tips indicating 95% CI. IV: inverse variance.

There was low certainty evidence from 7 (30%) trials [30,46,49,50,52,54,61] with 1575 participants that wearable activity tracker–based interventions significantly increased physical activity time in older adults compared to usual care immediately after intervention completion (SMD=0.28, 95% CI 0.10 to 0.47; *I*^2^=64%; *P*=.003; Figure 2A [30,31,33,46,49,50,52,54,57,61]; Table 2). However, there was moderate certainty evidence from 6 trials [30,31,33,46,54,57] involving 1325 participants that the wearable activity tracker–based interventions did not show a notable superiority over the conventional interventions in boosting physical activity in older adults immediately after intervention completion (SMD=0.11, 95% CI −0.08 to 0.30; *I*^2^=60%; *P*=.24; Figure 2A; Table 2).

**Table 2:** Certainty of evidence using Grading of Recommendations Assessment, Development, and Evaluation.

Follow-up and comparison		Outcome	Risk of bias	Inconsistency	Indirectness	Imprecision	Publication bias	Participants, N	SMD^a^ or MD^b^ (95% CI)	Certainty
**Immediate**										
	Usual care	Physical activity time	Serious^c^	Serious^d^	Not serious	Not serious	Not serious	1575 (7 RCTs^e^)	0.28 (0.10 to 0.47)	Low
	Conventional intervention	Physical activity time	Not serious	Serious^d^	Not serious	Not serious	Not serious	1325 (6 RCTs)	0.11 (−0.08 to 0.30)	Moderate
	Usual care	Daily step count	Not serious	Serious^d^	Not serious	Not serious	Not serious	2276 (15 RCTs)	0.58 (0.33 to 0.83)	Moderate
	Usual care	Daily sedentary time	Serious^c^	Not serious	Not serious	Not serious	Not serious	1391 (6 RCTs)	−1.56 (−10.88 to 7.76)	Moderate
	Conventional intervention	Daily sedentary time	Serious^c^	Not serious	Not serious	Not serious	Not serious	970 (3 RCTs)	13.95 (−1.03 to 28.93)	Moderate
**Short-term**										
	Usual care	Physical activity time	Serious^c^	Serious^d^	Not serious	Not serious	Not serious	1096 (4 RCTs)	0.20 (−0.03 to 0.42)	Low
	Conventional intervention	Physical activity time	Serious^c^	Serious^d^	Not serious	Not serious	Not serious	427 (3 RCTs)	0.13 (−0.31 to 0.58)	Low
	Usual care	Daily step count	Serious^c^	Not serious	Not serious	Not serious	Not serious	1040 (4 RCTs)	0.23 (0.11 to 0.36)	Moderate
	Usual care	BMI	Serious^c^	Not serious	Not serious	Not serious	Not serious	1590 (6 RCTs)	0.40 (−0.08 to 0.89)	Moderate
	Usual care	Body fat	Serious^c^	Not serious	Not serious	Not serious	Not serious	1013 (4 RCTs)	0.67 (−0.54 to 1.87)	Moderate
	Usual care	Timed up and go test	Not serious	Serious^d^	Not serious	Serious^f^	Not serious	209 (3 RCTs)	0.14 (−0.87 to 1.16)	Low
	Usual care	Chair stand test	Not serious	Not serious	Not serious	Serious^f^	Not serious	164 (3 RCTs)	−0.31 (−0.62 to 0)	Moderate

^a^SMD: standard mean difference.

^b^MD: mean difference.

^c^Downgrade due to the low methodological quality: PEDro (Physiotherapy Evidence Database) score <6.

^d^Downgrade due to large heterogeneity: *I*^2^ statistics >50%.

^e^RCT: randomized controlled trial.

^f^Downgrade due to pooled sample sizes: n<300.

At the short-term follow-up, there was no significant difference in the effectiveness of wearable activity tracker–based interventions in promoting physical activity among older adults compared to usual care (SMD=0.20, 95% CI −0.03 to 0.42; *I*^2^=62%; *P*=.09) or conventional interventions (SMD=0.13, 95% CI −0.31 to 0.58; *I*^2^=79%; *P*=.55; Figure 2B [30,46,49,50,57]). The quality of evidence supporting these findings was assessed as “low” (Table 2).

#### Daily Step Count

**Figure 3:**
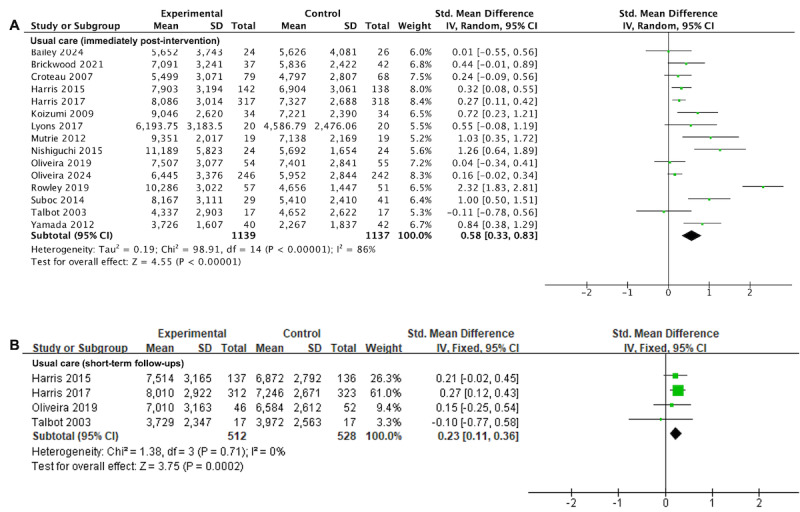
Forest plots for wearable activity tracker–based interventions compared with usual care in daily step count at (A) immediately after intervention and (B) short-term follow-ups. Green squares indicate standardized mean differences and mean differences, with larger squares reflecting greater weight; horizontal lines indicate 95% CI; and black diamonds indicate pooled effect estimates, with right and left tips indicating 95% CI. IV: inverse variance.

The pooled data from 15 (65%) trials [32,33,47-50,52,53,55,56,58-60,62,63] encompassing 2276 participants, indicated a significant association between wearable activity tracker–based interventions and higher daily step count compared with usual care immediately after intervention completion with moderate certainty evidence (SMD=0.58, 95% CI 0.33 to 0.83; *I*^2^=86%; *P*<.001; Figure 3A [32,33,47-50,52,53,55,56,58-60,62,63]; Table 2). Given the absence of noticeable asymmetry in the funnel plot (Multimedia Appendix 3) and the nonsignificant result from Egger test (*P*=.23), it suggested that publication bias is unlikely to have influenced the analysis of daily step count.

At the short-term follow-up, there was moderate certainty evidence from 4 trials [32,49,50,60] with 1040 participants that wearable activity tracker–based interventions also led to a significant increase in daily step count (SMD=0.23, 95% CI 0.11 to 0.36; *I*^2^=0%; *P*<.001; Figure 3B [32,49,50,60]; Table 2).

#### Daily Sedentary Time

**Figure 4:**
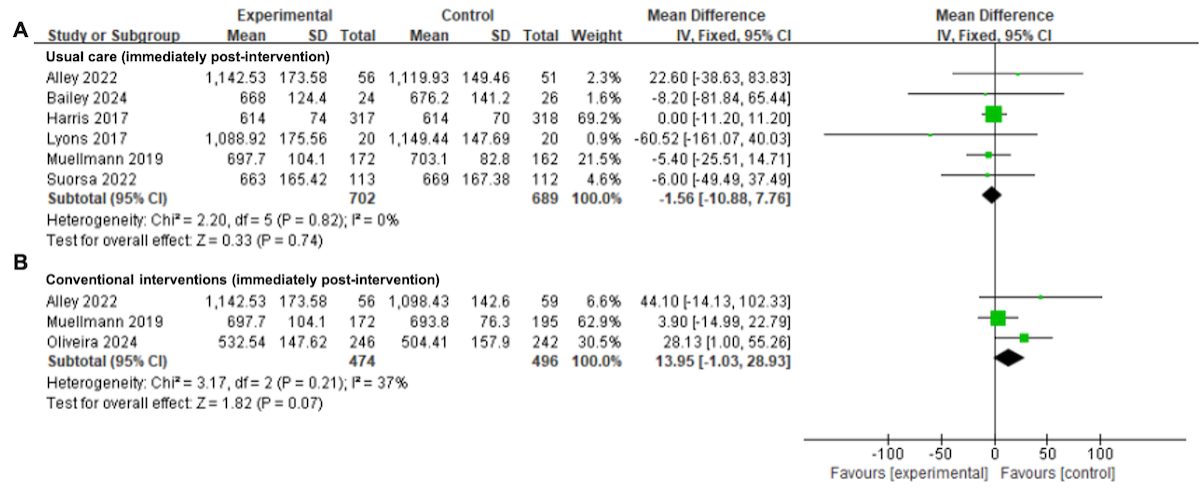
Forest plots for wearable activity tracker–based interventions compared with (A) usual care and (B) conventional interventions in daily sedentary time at immediate postintervention. Green squares indicate standardized mean differences and mean differences, with larger squares reflecting greater weight; horizontal lines indicate 95% CI; and black diamonds indicate pooled effect estimates, with right and left tips indicating 95% CI. IV: inverse variance.

A meta-analysis of 6 (26%) trials [34,46,49,53,54,63] involving 1391 participants revealed no significant difference in the effectiveness of wearable activity tracker–based interventions on decreasing daily sedentary time compared to usual care immediately after intervention completion (MD=−1.56, 95% CI −10.88 to 7.76; *I*^2^=0%; *P*=.74; Figure 4A [34,46,49,53,54,63]). The quality of evidence supporting this finding was rated as “moderate” (Table 2). Similarly, there was moderate certainty evidence from 3 trials [33,46,54] involving 970 participants that the wearable activity tracker–based interventions did not show a significant difference compared to the conventional interventions in improving daily sedentary time among older adults immediately after intervention completion (MD=13.95, 95% CI −1.03 to 28.93; *I*^2^=37%; *P*=.07; Figure 4B [33,46,54]; Table 2).

### Effects on Body Composition

#### BMI Analysis

**Figure 5:**
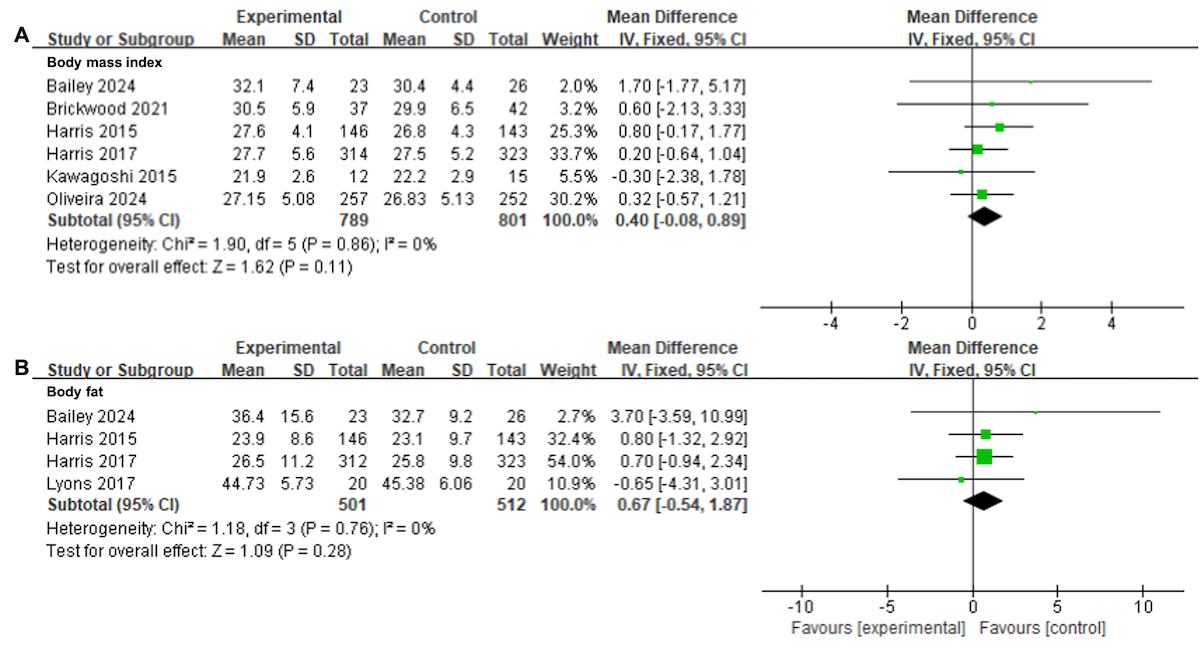
Forest plots for wearable activity tracker–based interventions compared with usual care in (A) BMI and (B) body fat. Green squares indicate standardized mean differences and mean differences, with larger squares reflecting greater weight; horizontal lines indicate 95% CI; and black diamonds indicate pooled effect estimates, with right and left tips indicating 95% CI. IV: inverse variance.

In total, 6 (26%) studies [33,47,49-51,63] involving 1590 participants provided moderate certainty evidence that there was no significant difference between the wearable activity tracker–based interventions and usual care in improving BMI (MD=0.40, 95% CI −0.08 to 0.89; *I*^2^=0%; *P*=.11; Figure 5A [33,47,49-51,63]; Table 2).

#### Body Fat

On the basis of 4 (17%) studies [49,50,53,63], including 1013 people, there was moderate certainty evidence that the wearable activity tracker–based interventions did not have a greater impact on body fat than usual care (MD=0.67, 95% CI −0.54 to 1.87; *I*^2^=0%; *P*=.28; Figure 5B [49,50,53,63]; Table 2).

### Effects on Physical Function

#### Timed Up and Go Test

**Figure 6:**
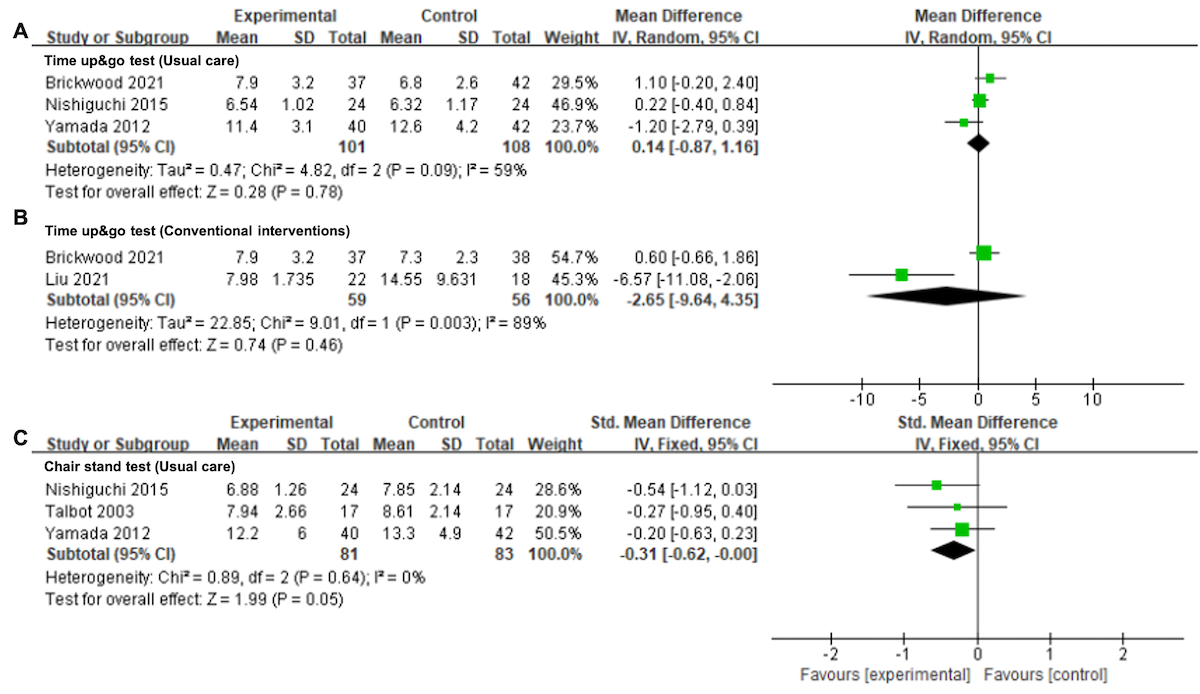
Forest plots for wearable activity tracker–based interventions compared with (A) usual care and (B) conventional interventions in timed up and go test and compared with (C) usual care in chair stand test. Green squares indicate standardized mean differences and mean differences, with larger squares reflecting greater weight; horizontal lines indicate 95% CI; and black diamonds indicate pooled effect estimates, with right and left tips indicating 95% CI. IV: inverse variance.

There was low certainty evidence from 3 (13%) trials [47,56,62] with 209 participants that older adults who underwent wearable activity tracker–based interventions did not show significantly better performance on the timed up and go test than those who underwent usual care (MD=0.14, 95% CI −0.87 to 1.16; *I*^2^=59%; *P*=.78; Figure 6A [47,56,62]; Table 2). In addition, there was no significant difference between the use of the wearable activity tracker–based interventions and conventional interventions in promoting the timed up and go test among older adults (MD=−2.65, 95% CI −9.64 to 4.35; *I*^2^=89%; *P*=.46; Figure 6B [31,47]; Table 2).

#### Chair Stand Test

The pooled data from 3 (13%) trials [56,60,62] involving 164 participants, suggested a trend toward improved performance in the chair stand test following wearable activity tracker–based interventions with moderate certainty evidence, which did not achieve statistical significance (MD=−0.31, 95% CI −0.62 to 0; *I*^2^=0%; *P*=.05; Figure 6C [56,60,62]; Table 2).

## Discussion

### Principal Findings

In this systematic review and meta-analysis, we conducted a pooled analysis to evaluate the impact of wearable activity tracker–based interventions on physical activity, body composition, and physical function among community-dwelling older adults. The findings from this study suggest that such interventions might be more efficacious in enhancing physical activity than usual care, particularly in terms of physical activity time supported by low certainty and daily step count supported by moderate certainty, with the most notable improvements observed immediately after intervention. However, significant effects on body composition or physical function were not detected, as supported by low to moderate certainty evidence. Nevertheless, wearable activity tracker–based interventions seemed to be at least as effective as conventional interventions, such as behavior change techniques, tailored exercises, and prescribed physical activity, as supported by low to moderate certainty evidence. Moreover, our findings indicated a potential for the sustained positive impact of wearable activity tracker use on daily step count during short-term follow-ups, with moderate certainty.

Using wearable activity trackers, the observed improvements compared with usual care in physical activity time and daily step count are encouraging. This finding suggests that wearable activity trackers have the potential to act as valid motivators for older adults to incorporate regular physical activity into their everyday routines, due to timely feedback, self-monitoring, and goal setting. However, no significant changes were detected in daily sedentary time among older adults following wearable activity tracker–based interventions, which aligns with the findings of previous meta-analysis [64,65]. This may be caused by the different regulatory processes between intentional behaviors and habitual behaviors [66,67]. Intentional behaviors are typically enhanced through strategies such as monitoring, feedback, and rewards, which are commonly incorporated into wearable activity tracker–based designs [68]. By providing immediate and positive reinforcement, these features serve to activate the desired behaviors, such as activity time and step count. However, such interventions usually place less emphasis on modifying habitual behaviors, such as sedentary patterns, which are automatic and require comprehensive strategies to effect change. Thus, additional trials are needed to enhance wearable activity tracker–based interventions to effectively change habitual activities.

Despite these positive outcomes in physical activity, wearable activity tracker–based interventions did not yield significant effects on body composition and physical function. This finding suggests that increasing physical activity alone may not be sufficient to elicit measurable changes in these outcomes, particularly in older populations influenced by factors such as diet, psychological state, and functional limitations. For body composition, measurable changes often require both increased physical activity and dietary modifications, as exercise alone may not effectively alter energy balance or muscle mass. Similarly, the lack of significant improvements in physical function may be due to the nature of wearable activity tracker–based interventions, which primarily encourage general movement rather than structured resistance or balance training. Physical function in older adults is often influenced by muscle strength, coordination, and neuromuscular control, which may not be adequately addressed through physical activity time or step count increases. Furthermore, preexisting limitations, fear of injury, and individual variability may further restrict these outcomes. Given these considerations, future interventions may need more comprehensive elements to maximize improvements in body composition and physical function. In addition, future studies should explore the impact of wearable activity trackers on a broader range of health outcomes in older adults, including cognitive function, fall prevention, sleep quality, mental health, and social engagement. Such research could provide a more comprehensive understanding of the multifaceted benefits of wearable devices and help identify strategies for optimizing their use to improve overall well-being in older populations.

Interestingly, wearable activity tracker–based interventions have been shown to be at least as effective as conventional interventions, such as behavior change techniques, tailored exercises, and prescribed physical activity. However, it is important to note that in many of the included studies, wearable activity trackers were implemented as adjuncts to conventional interventions, rather than as standalone strategies. Thus, the observed effectiveness likely reflects the combined impact of activity trackers and conventional intervention components, rather than the independent effect of the devices. The absence of a significant additional benefit from activity tracker–based interventions compared to conventional interventions alone suggests that these devices may not inherently amplify the efficacy of existing interventions. However, compared to conventional face-to-face and counseling phone interventions, wearable activity trackers offer potential advantages, including being less resource-intensive, more scalable, and providing a practical and personalized approach. These features may enhance participant engagement or adherence to conventional interventions. Furthermore, wearable devices have the potential to bridge gaps in conventional interventions by providing objective measures of adherence and progress, which are critical for evaluating long-term outcomes. Nevertheless, future research is needed to assess the effectiveness of activity trackers as standalone tools and to explore the specific mechanisms through which they may influence behavior change. This will help clarify their potential role in promoting physical activity, particularly among older adults.

The immediate postintervention improvements observed in our study are particularly noteworthy, as they suggest that the use of wearable activity trackers can have a rapid and positive impact on older adults, but not on sustained maintenance. This may be attributed to the short duration of intervention in the included trials, most of which were 12 weeks. Making specific evident lifestyle modifications in a limited period and maintaining these changes in behavior over the long term is challenging, particularly for older adults. Hence, future research should also focus on extending intervention periods to assess whether longer durations can sustain and amplify the benefits observed during shorter interventions. Investigating the long-term effects of wearable activity trackers is essential to understanding their potential to support sustained behavioral changes and achieve clinically meaningful outcomes. Such studies would help determine the optimal duration of interventions and whether prolonged use enhances adherence, physical activity levels, and broader health benefits. It would also be valuable to explore tailored approaches that consider individual differences in technological literacy, motivation, and health conditions to optimize the design and implementation of interventions for older adults.

### Strengths and Limitations

To our knowledge, this is an up-to-date summary evaluating the wearable activity trackers in community-dwelling older adults. Our focus extends beyond merely physical activity to evaluate body composition and physical functionality. We conducted this systematic review following PRISMA guidelines (Multimedia Appendix 4) and prospectively registered in PROSPERO. In addition, most of the included trials were of high quality, with a mean PEDro score of 6. We also conducted comparisons between interventions based on wearable activity trackers and other interventions, which included both active and passive control groups, providing a crucial perspective for understanding the comprehensive impact of wearable technology. Despite these strengths, this review has certain limitations that may be addressed in future research. First and most significantly, the inclusion of participants aged ≥55 years, rather than the traditional threshold of participants aged 60 years, may limit the extrapolation of our findings to older populations. While this criterion aligns with some aging-related research, it remains a limitation given the increasing life expectancy and shifting age-related health benchmarks. Furthermore, the relatively short duration of intervention in the included trials poses a significant limitation to understanding the long-term efficacy of wearable activity tracker–based interventions. While immediate postintervention improvements are promising, these short intervention periods may not provide sufficient time for older adults to establish and maintain substantial lifestyle changes. In addition, the potential bias from self-reported physical activity may affect the results, as participants may overestimate or underreport their activity levels. Finally, differences in the intervention components across the studies included in our analysis may have contributed to the observed heterogeneity in outcomes, making it challenging to draw consistent conclusions.

### Conclusions

The findings of this review suggest that the wearable activity tracker–based interventions were particularly effective at enhancing physical activity among community-dwelling older adults, as evidenced by increased physical activity time with low certainty and daily step counts with moderate certainty, especially immediately after an intervention. However, these interventions did not have a significant impact on body composition or physical function, with low to moderate certainty. It is important to note that the positive effects were more pronounced when compared against usual care, rather than against conventional interventions, with low to moderate certainty. In addition, this intervention showed moderate certainty evidence for improving daily step count, supporting its sustained impact during short-term follow-up. Given these findings, there is a clear need for future research to focus on the short- or long-term effects of such interventions and explore strategies to maximize their impact on a broader range of health outcomes.

## Data Availability

The datasets generated and analyzed during this study are available from the corresponding author on reasonable request.

## Appendix

Multimedia Appendix 1 Search strategy for the databases PubMed, Embase, CENTRAL, and Web of Science.

Multimedia Appendix 2 Methodological quality and reporting of eligible studies.

Multimedia Appendix 3 Funnel plot for daily step count between wearable activity tracker–based interventions and usual care.

Multimedia Appendix 4 PRISMA (Preferred Reporting Items for Systematic Reviews and Meta-Analyses) checklist.

## Abbreviations

MD: mean difference
PEDro: Physiotherapy Evidence Database
PRISMA: Preferred Reporting Items for Systematic Reviews and Meta-Analyses
RCT: randomized controlled trial
SMD: standardized mean difference
