# Correction: Wearable Activity Tracker–Based Interventions for Physical Activity, Body Composition, and Physical Function Among Community-Dwelling Older Adults: Systematic Review and Meta-Analysis of Randomized Controlled Trials

**DOI:** 10.2196/79133

**Published:** 2025-08-29

**Authors:** Ran Li, Yangan Li, Lu Wang, Lijuan Li, Chenying Fu, Danrong Hu, Quan Wei

**Affiliations:** 1 Department of Rehabilitation Medicine Center and Institute of Rehabilitation Medicine, West China Hospital, Sichuan University Chengdu, Sichuan China; 2 Key Laboratory of Rehabilitation Medicine in Sichuan Province Chengdu, Sichuan China; 3 National Clinical Research Center for Geriatrics, West China Hospital, Sichuan University Chengdu, Sichuan China; 4 Aging and Geriatric mechanism laboratory, West China Hospital, Sichuan University Chengdu, Sichuan China

In “Wearable Activity Tracker–Based Interventions for Physical Activity, Body Composition, and Physical Function Among Community-Dwelling Older Adults: Systematic Review and Meta-Analysis of Randomized Controlled Trials” (J Med Internet Res 2025;27:e59507) several errors were noted.

In Table 1 of the “Results” section, the following rows:

- *Row 7 Column “Study design”: 2-arm RCT*
- *Row 13 Column “Follow-up”: 12wk and 48wk*
- *Row 20 Column “Duration”: 48mo*
- *Row 23 Column “Sex, n (%): Male, 34 (57); Female, 26 (43)*

Have been changed to read as follows:

- *Row 7 Column “Study design”: 3-arm RCT*
- *Row 13 Column “Follow-up”: 12wk and 24wk*
- *Row 20 Column “Duration”: 48wk*
- *Row 23 Column “Sex, n (%): Male, 16 (27); Female, 44 (73)*

Additionally, after the final sentence of “Study Selection & Characteristics” subsection in “Methods” section, the following sentence has been added before Table 1:

Several study durations originally reported in months were approximated using the convention of 1 month = 4 weeks for consistency in presentation. This approximation does not affect the timing of outcome assessments or any statistical analyses.

The corrections will appear in the online version of the paper on the JMIR Publications website, together with the publication of this correction notice. Because this was made after submission to PubMed, PubMed Central, and other full-text repositories, the corrected article has also been resubmitted to those repositories.

