# Supplementary material for: Wearable Activity Tracker–Based Interventions for Physical Activity, Body Composition, and Physical Function Among Community-Dwelling Older Adults: Systematic Review and Meta-Analysis of Randomized Controlled Trials
Source: J Med Internet Res. 2025 Apr 3;27:e59507. doi: 10.2196/59507 (PMC12006780; doi:10.2196/59507)
Supplement: Multimedia Appendix 1 [file jmir_v27i1e59507_app1.docx]

**Multimedia Appendix 1**

**Search strategy for Pubmed, Embase, CENTRAL and Web of science.**

**Search strategy for Pubmed <January 2025>**

#1 (((((((Fitness Tracker[Title/Abstract]) OR (Physical Fitness Tracker[Title/Abstract])) OR (Activity Tracker[Title/Abstract])) OR (Personal Fitness Tracker[Title/Abstract])) OR (Pedometer[Title/Abstract])) OR (Wearable Tracker[Title/Abstract])) OR (Wearable Device[Title/Abstract])) OR (Electronic tracker[Title/Abstract]) 7447

#2 ((((Fitbit[Title/Abstract]) OR (Garmin[Title/Abstract])) OR (smartwatch[Title/Abstract])) OR (bracelet[Title/Abstract])) OR (Jawbone[Title/Abstract]) 4461

#3 (((old[Title/Abstract]) OR (older[Title/Abstract])) OR (elder[Title/Abstract])) OR (elderly[Title/Abstract]) 2135515

#4 (randomized controlled trial[pt] OR controlled clinical trial[pt] OR randomized[tiab] OR placebo[tiab] OR clinical trials as topic[mesh:noexp] OR randomly[tiab] OR trial[ti] NOT (animals[mh] NOT humans [mh])) 1539708

#5 (#1 OR #2) AND (#3) AND (#4) 335

**Search strategy for Embase <January 2025>**

#1 ('Fitness Tracker' or 'Physical Fitness Tracker' or 'Activity Tracker' or 'Personal Fitness Tracker' or 'Pedometer' or 'Wearable Tracker' or 'Wearable Device' or 'Electronic tracker').mp. 14958

#2 (Fitbit or Garmin or smartwatch or bracelet or Jawbone).mp. 6363

#3 (old or older or elder or elderly).mp. 3268074

#4 (randomized controlled trial not (animals not humans)).mp. 1151471

#5 #1 or #2 19790

#6 #3 and #4 and #5 468

**Search strategy for Cochrane Central Register of Controlled Trials <January 2025>**

#1 ('Fitness Tracker' or 'Physical Fitness Tracker' or 'Activity Tracker' or 'Personal Fitness Tracker' or 'Pedometer' or 'Wearable Tracker' or 'Wearable Device' or 'Electronic tracker').mp. 3402

#2 (Fitbit or Garmin or smartwatch or bracelet or Jawbone).mp. 1837

#3 (old or older or elder or elderly).mp. 211738

#4 (randomized controlled trial not (animals not humans)).mp. 685568

#5 #1 or #2 4846

#6 #3 and #4 and #5 402

**Search strategy for Web of science <January 2025>**

#1 (TI=((Fitness Tracker OR Physical Fitness Tracker OR Activity Tracker OR Personal Fitness Tracker OR Pedometer OR Wearable Tracker OR Wearable Device OR Electronic tracker))) OR AB=((Fitness Tracker OR Physical Fitness Tracker OR Activity Tracker OR Personal Fitness Tracker OR Pedometer OR Wearable Tracker OR Wearable Device OR Electronic tracker)) 253876

#2 (TI=((Fitbit OR Garmin OR smartwatch OR bracelet OR Jawbone))) OR AB=((Fitbit OR Garmin OR smartwatch OR bracelet OR Jawbone)) 53282

#3 (TI=((old OR older OR elder OR elderly))) OR AB=((old OR older OR elder OR elderly)) 3848212

#4 TS=(randomized controlled trial) 877708

#5 #1 OR #2 293724

#6 #3 AND #4 AND #5 433
