# Supplementary material for: Wearable Activity Tracker–Based Interventions for Physical Activity, Body Composition, and Physical Function Among Community-Dwelling Older Adults: Systematic Review and Meta-Analysis of Randomized Controlled Trials
Source: J Med Internet Res. 2025 Apr 3;27:e59507. doi: 10.2196/59507 (PMC12006780; doi:10.2196/59507)
Supplement: Multimedia Appendix 2 [file jmir_v27i1e59507_app2.docx]

**Multimedia Appendix 2**

| **Methodological quality and reporting of eligible studies.** | | | | | | | | | | | |
| --- | --- | --- | --- | --- | --- | --- | --- | --- | --- | --- | --- |
| **Study** | **PEDro Scale Items** | | | | | | | | | | **PEDro Score (0-10)** |
|  | **Random allocation** | **Concealed allocation** | **Baseline comparability** | **Participant blinding** | **Therapist blinding** | **Assessor blinding** | **Adequate follow-up** | **Intent-to-treat analysis** | **Between-group comparison** | **Point estimate and variability** |  |
| Alley [46] 2022 | Y | N | N | N | N | N | N | N | Y | Y | 3 |
| Armit [30] 2009 | Y | N | Y | N | N | N | Y | Y | Y | Y | 6 |
| Bailey [63] 2024 | Y | Y | Y | N | N | N | N | N | Y | Y | 5 |
| Brickwood [47] 2021 | Y | Y | Y | N | N | N | N | Y | Y | Y | 6 |
| Croteau [48] 2007 | Y | Y | N | N | N | N | N | N | Y | Y | 3 |
| Harris [50] 2015 | Y | N | Y | N | N | N | Y | N | Y | Y | 5 |
| Harris [49] 2017 | Y | N | N | N | N | N | N | N | Y | Y | 3 |
| Kawagoshi [51] 2015 | Y | N | Y | N | N | N | N | N | Y | Y | 4 |
| Koizumi [52] 2009 | Y | N | Y | N | N | N | Y | N | Y | Y | 5 |
| Liu [31] 2021 | Y | Y | Y | N | N | Y | Y | N | Y | Y | 7 |
| Lyons [53] 2017 | Y | Y | Y | N | N | N | Y | Y | Y | Y | 7 |
| Muellmann [54] 2019 | Y | Y | Y | N | N | Y | N | N | Y | Y | 6 |
| Mutrie [55] 2012 | Y | Y | Y | N | N | N | Y | Y | Y | Y | 7 |
| Nishiguchi [56] 2015 | Y | N | Y | N | N | Y | Y | N | Y | Y | 6 |
| Oliveira [33] 2024 | Y | Y | Y | N | N | Y | Y | Y | Y | Y | 8 |
| Oliveira [32] 2019 | Y | Y | Y | N | N | Y | Y | Y | Y | Y | 8 |
| Patel [57] 2013 | Y | N | Y | N | N | N | N | N | Y | Y | 4 |
| Rowley [58] 2019 | Y | N | Y | N | N | N | N | N | Y | Y | 4 |
| Suboc [59] 2014 | Y | N | Y | N | N | Y | Y | Y | Y | Y | 7 |
| Suorsa [34] 2022 | Y | Y | Y | N | N | N | Y | Y | Y | Y | 7 |
| Talbot [60] 2003 | Y | N | Y | N | N | N | Y | N | Y | Y | 5 |
| Yamada [62] 2012 | Y | Y | Y | N | N | Y | Y | N | Y | Y | 7 |
| Yuenyongchaiwat and Akekawatchai [61] 2022 | Y | N | Y | N | N | Y | Y | Y | Y | Y | 7 |

PEDro = physiotherapy evidence database; Y = yes; N = no.
