# Supplementary material for: Wearable Activity Tracker–Based Interventions for Physical Activity, Body Composition, and Physical Function Among Community-Dwelling Older Adults: Systematic Review and Meta-Analysis of Randomized Controlled Trials
Source: J Med Internet Res. 2025 Apr 3;27:e59507. doi: 10.2196/59507 (PMC12006780; doi:10.2196/59507)
Supplement: Multimedia Appendix 3 [file jmir_v27i1e59507_app3.docx]

**Multimedia Appendix 3**


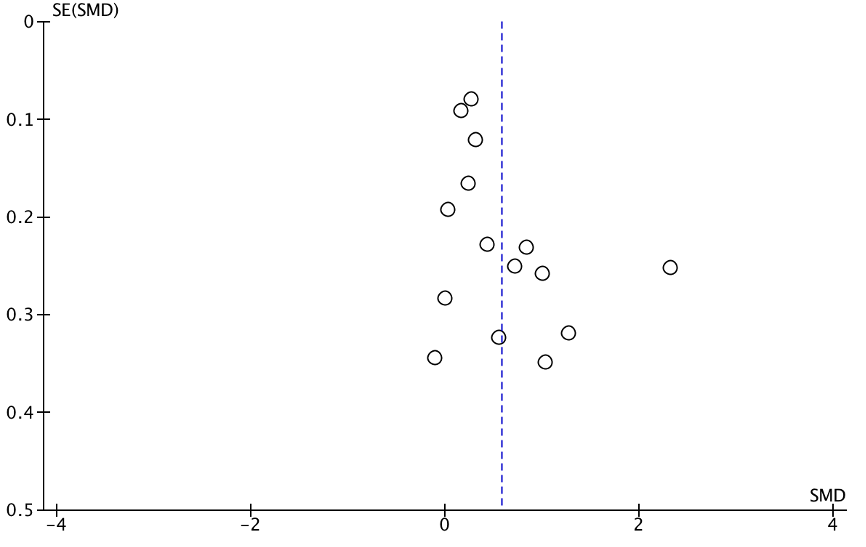


**Funnel plot for daily step count between wearable activity tracker-based interventions and usual care.**
